# Properties and Application of a Partially Purified Thermoalkali Stable Xylanase from *Cellulosimicrobium* sp. MTCC 10645 in Kraft Pulp Bleaching

**DOI:** 10.5402/2013/872325

**Published:** 2012-06-04

**Authors:** Rajashri D. Kamble, Anandrao R. Jadhav

**Affiliations:** ^1^Department of Biotechnology Engineering, Tatyasaheb Kore Institute of Engineering and Technology, Warananagar, Kolhapur, Maharashtra 416113, India; ^2^Department of Microbiology, KRP Kanya Mahavidyalaya, Islampur, Sangli, Maharashtra 415414, India

## Abstract

The most promising application of xylanases (E.C. 3.2.1.8) is in the prebleaching of kraft pulp. The present paper reports bleaching effects of a thermoalkali stable xylanase from *Cellulosimicrobium *sp. MTCC 10645. The bacterium produced thermo-alkali stable xylanase in a basal medium supplemented with wheat bran (1% w/v), which was optimally active at pH 7.0 and 50°C. The xylanase was stable at temperature 50°C for 1 h and retained up to 86% of the activity. The xylanase was stable in a broad pH range of 6.0–11.0 for 1 h at 50°C. Metal ions Ca^+2^, Hg^+2^, and Pb^+2^ were inhibitory for xylanase retaining 72.3%, 35.07% and 36.7% relative activity at 10 mM concentration, whereas Fe^+2^, Cu^+2^, Mn^+2^, Na^+2^, Co^+2^, and Zn^+2^ were inducers at concentrations of 5 mM and 10 mM. The enzyme exhibited greater binding affinity exclusively for xylans but not for avicel, CMC, cellobiose, starch, or p-nitrophenyl xylopyranoside. Parachloromercuric benzoate and iodoacetamide were found stimulatory, while potassium permanganate, cysteine, and cystine markedly reduced the activity. The xylanase dose of 2.0 U/g dry weight pulp of 10% consistency gave optimum bleach boosting of kraft pulp at pH 8.0 and temperature 50°C for 5 h reaction time.

## 1. Introduction

The most promising application of xylanases (E.C. 3.2.1.8) is in the prebleaching of kraft pulp. The pulp and paper industry is modifying its pulping, bleaching, and effluent treatment technologies to reduce the environmental impact of mill effluents. Prebleaching of kraft pulps with xylanases lowers chlorine charges, which reduce chloroorganic discharges [1–3]. Tremblay and Archibald reported the delignification of unbleached softwood and hardwood kraft pulps [4]. Thus reducing the Cl_2_ required to achieve a given degree of bleaching [3, 5–7]. The public concern on the impact of pollutants from paper and pulp industries, which use chlorine as the bleaching agent act as strong driving force in developing biotechnology aided techniques for novel bleaching that is biobleaching [8, 9]. The occurrence of cellulase contamination is posing a major threat in applying the xylanases in biobleaching. The cellulases easily result in the hydrolysis of cellulose, which should be the main recovered product in paper industry. However, the enzyme preparations from microorganisms producing higher levels of xylanases with no cellulase activity can be applied in paper industry because the loss of pulp viscosity is at minimum level [10]. Xylanases have been reported from bacteria, fungi, actinomycetes, and yeasts [11–14]. The use of abundantly available and cost-effective agricultural residues to achieve higher xylanase yields and simple and rapid purification procedures provide suitable means to reduce the manufacturing cost of biobleached paper. The present paper reports partial purification, properties, and the bleaching effects of an thermo-alkali stable xylanase from *Cellulosimicrobium* sp. MTCC 10645.

## 2. Materials and Methods

### 2.1. Microorganism and Culture Conditions

*Cellulosimicrobium* sp. MTCC 10645 was isolated from the compost sample collected at Kolhapur (M.S., India). The culture was purified and maintained on xylan agar (pH 7.0) supplemented with 0.1% oat spelt xylan. The xylanase production was carried out in 250 mL Erlenmeyer flasks, each containing 18 mL basal salt solution supplemented with 10 g of wheat bran (1 : 1.8 moisture ratio) in static conditions [15]. Each flask was inoculated with 2% bacterial inoculum and incubated at 40°C for 7 days. 

### 2.2. Assays

Xylanase activity was determined by measuring the release of reducing sugars from birchwood xylan (1% w/v) using the dinitrosalicylic acid method [16]. One unit of xylanase activity was defined as the amount of enzyme that liberates 1 *μ*mol of reducing sugars equivalent to xylose per minute under the assay conditions (50°C, 50 mM sodium phosphate buffer pH 7.0). Total soluble protein was measured using bovine serum albumin as a standard [17].

### 2.3. Partial Purification and Properties of Xylanase

The cell free supernatant (75 mL) was precipitated using fractional (35–80%) ammonium sulphate saturation. The precipitate was dialysed and the protein thus obtained was treated as partially purified enzyme and used for further experiments. The thermal stability was determined by incubating the equal volume of enzyme solution at temperatures ranges between 30°C–80°C for 4 h. Optimal pH for xylanase activity was determined using different buffers 50 mM sodium phosphate (pH 6.0, 7.0), 50 mM Tris HCl (pH 8.0, 9.0), 50 mM carbonate bicarbonate buffer (pH 10.0) and 50 mM glycineNaOH buffer (pH 11.0). The pH stability was determined by incubating the equal volume of enzyme solution with different buffers ranging from 6.0–11.0 at 50°C and the residual activities were determined after 1 h. The effect of various metal ions solution of chloride salts of either Ca^+2^, Hg^+2^, Fe^+2^, Cu^+2^, Mn^+2^, Na^+2^, Co^+2^, Pb^+2^, and Zn^+2^ at 1 mM, 5 mM, and 10 mM concentration on enzyme activity was determined by incubating the enzyme with respective compounds at room temperature for 1 h followed by determination of residual activities under standard assay conditions. Substrate specificity of the xylanase was studied by using 1% xylan, cellobiose, starch, carboxy methyl cellulose (CMC), *p*-nitrophenyl xylopyranoside, and avicel as substrates. Effect of additives like parachloromercuric benzoate, iodoacetamide, potassium permanganate, cysteine, and cystine (0.5 mM) was tested on xylanase activity.

### 2.4. Xylanase Bleaching of Kraft Pulp

The enzymatic bleaching studies were performed at pH 8.0 at 50°C, unless otherwise mentioned. The optimization of enzyme dose, pulp consistency and reaction time was carried out by treating the pulp with different doses of xylanase from *Cellulosimicrobium* sp. MTCC 10645 at 2 U/g, 5 U/g, 10 U/g, and 25 U/g oven dried pulp of consistency 3.0%, 5% and 10% for variable time intervals 3 h and 5 h. The chemical characteristics of Kraft pulp: kappa number (T 236 cm-85), release of reducing sugars, release of phenolic compounds (A237 nm and 280 nm), and hydrophobic compounds (A465 nm) were determined [16, 18, 19].

## 3. Results and Discussion

### 3.1. Xylanase Production

The test organism was isolated from compost sample collected at Kolhapur (MS, India). It was identified as *Cellulosimicrobium* sp. and deposited with Microbial Type Culture Collection no. 10645 at IMTECH, Chandigarh, India. Xylanase production was monitored in *Cellulosimicrobium* sp. MTCC 10645 grown for 7 days in static conditions on wheat bran. Xylanase activity appeared after day 1 and reached maximum (960 U/mL) after day 2 at pH 7.0 and 40°C. 

### 3.2. Properties of Partially Purified Xylanase

**Figure 1:**
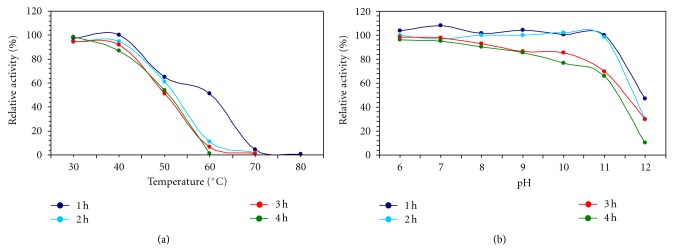
(a) Temperature stability profile of xylanase assayed at pH 7.0 for 4 h. (b) pH stability profile of xylanase assayed at 50°C for 4 h.

The extracellular xylanase from *Cellulosimicrobium* sp. MTCC 10645 was partially purified at 80% (NH_4_)_2_SO_4_ saturation with a yield of 52.03% and a purification fold of 16.0. The partially purified xylanase from *Cellulosimicrobium *sp. MTCC 10645 was optimally active at 50°C and pH 7.0 (Figures 1(a) and 1(b)). 

The xylanase was stable at temperature 50°C for 1 h and retained up to 86% of the activity. Interestingly at 50°C relative activity was comparatively constant up to 4 h of incubation at pH 7.0. Reportedly, bacterial xylanases found more thermostable than fungal xylanases. Most of the thermostable xylanases are produced by mesophilic bacteria [15, 20]. Two xylanases which gave the highest activity at 50°C were purified cell-free extract of *Cephalosporium *sp. [21]. The xylanase obtained from *Cellulosimicrobium *sp. MTCC 10645 was stable in a broad pH range of 6.0–11.0 for 1 h at 50°C. It showed highest activity at pH 7.0 and found more stable at pH 7.0 even after 4 h of incubation. The most desirable characteristic was its steady stability at alkaline pH values. Mathrani and Ahring isolated a thermophilic and alkaliphilic xylanase from *Dictyoglomus* isolate and reported that almost 100% activity shown by the xylanase at pH 5.5 to 9.0 [22]. 

As shown in Table 1, metal ions Ca^+2^, Hg^+2^, and Pb^+2^ were inhibitory for *Cellulosimicrobium *sp., MTCC 10645 xylanase retaining 72.3%, 35.07%, and 36.7% relative activity at 10 mM concentration, while Fe^+2^, Cu^+2^, Mn^+2^, Na^+2^, Co^+2^, and Zn^+2^ were inducers at concentrations 5 mM and 10 mM. Sreenath and Joseph reported the stimulatory effect of Ca^+2^ and Na^+2^ on xylanases of *Streptomyces exfoliates* [23]. Gessesse reported the inhibitory activity of Pb^+2^ on xylanase at 1 mM concentration in an alkaliphilic *Bacillus *sp. with 8% residual activity [24].

The xylanase was active on oat spelt and birchwood xylans but not on avicel, CMC, cellobiose, starch, or *p*-nitrophenyl xylopyranoside. Purified xylanase was not active on avicel, CMC, cellobiose, starch, and *p*-nitrophenyl xylopyranoside even when the enzyme concentration was 5 times greater than used in normal assay at an incubation period of 20 minutes rather than 5 minutes suggesting that enzyme is a true xylanase. Similarly, xylanase with absolute substrate specificity was purified from *Trichoderma viride* [25]. Two endo-1,4-*β*-xylanases from *Irpex lacteus* (*Polyporus tulipiferae*) were purified, named xyl I and III which did not showed activity towards glycans such as starch, pachyman, and avicel (microcrystalline cellulose) but showed one twentieth activity towards carboxymethyl cellulose [26]. Additives parachloromercuric benzoate and iodoacetamide were found stimulatory, while potassium permanganate, cysteine, and cystine markedly reduced the enzyme activity. Similar observation was reported by Anthony et al. in case of high-molecular-weight cellulase free xylanase from alkali tolerant *Aspergillus fumigatus* AR1 [27]. Iodoacetamide was found to be stimulatory for xylanase activity at 0.5 mM and 1.0 mM concentrations. These compounds showed similar effect on xylanase isolated from *Thermomonospora *sp. [28].

### 3.3. Biobleaching of Kraft Pulp

**Figure 2:**
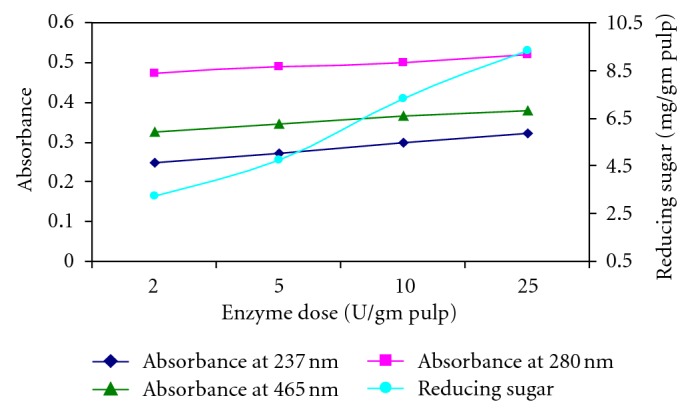
Optimization of xylanase dose for the enzymatic bleaching of kraft pulp at 50°C, pH 8.0 after 5 h.

The xylanase dose for biobleaching of kraft pulp at 50°C was optimized as 2.0 U/g of moisture free pulp (Figure 2). Reducing sugars released were 3.235 mg/g, 4.75 mg/g, 7.34 mg/g, and 9.321 mg/g of the pulp, when the enzyme dose given 2 U/g, 5 U/g, 10 U/g, and 25 U/g, respectively. At wavelengths 237 nm, 280 nm, and 465 nm, the absorbance was found to be increased from 0.247 to 0.321, 0.471 to 0.521, and 0.325 to 0.378 with increased dose of enzyme, respectively. Li et al. reported that the amount of reducing sugars released from wheat straw pulp by the xylanase isolated from *Thermomyces lanuginosus *CBS 288.54 was significantly greater with increasing time [29]. 

**Figure 3:**
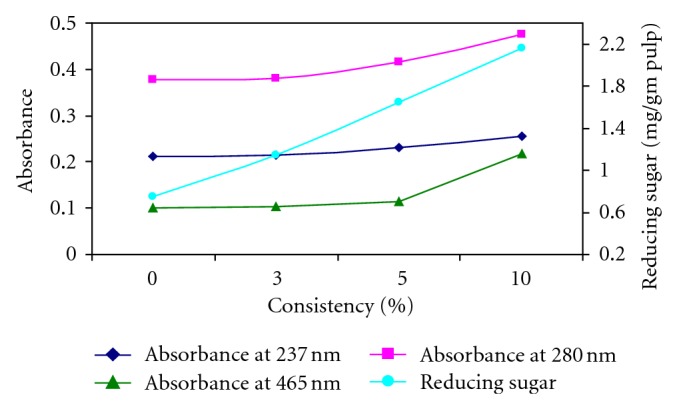
Optimization of consistency of pulp for the enzymatic bleaching of kraft pulp at 50°C, pH 8.0 after 5 h.

When kraft pulp was treated with xylanase from *Cellulosimicrobium *sp. MTCC 10645, the release of light absorbing material and reducing sugars increased with increase in consistency. Release of absorbing materials at 237 nm, 280 nm, and 465 nm can be correlated with release of lignin. The absorbance measured was highest at 280 nm wavelength as compared to 237 nm and 465 nm at 10% pulp consistency. At 237 nm wavelength, the absorbance measured was 0.215, 0.231, and 0.256 at 3%, 5%, and 10% consistency of pulp, respectively. At 0% pulp consistency, the absorbance measured was 0.211 at 237 nm wavelength. At 465 nm the absorbance measured was comparatively less 0.218 at 10% pulp consistency. The reducing sugars released were 0.75 mg/g, 1.15 mg/g, 1.65 mg/g, and 2.161 mg/g of pulp at 0%, 3%, 5%, and 10% consistency, respectively (Figure 3). Elegir et al. reported that the release of chromophores correlates well with total sugar release and this can be considered as a simple method to determine the efficacy of the enzyme treatment [30]. Suurnakki et al. reported that the decrease in the lignin content of the pulp did correlate with the degree of solubilization of carbohydrates [31]. In the xylanase treatment, pulp xylan was hydrolysed to soluble xylooligomers. The increase in absorbance at 280 nm as well as the increase in the liberation of reducing sugars indicated the effective action of xylanase on pulp. 

In the present study, the brightness increased from 30.13% (brightness of unbleached pulp) to 70.45% after the treatment of the kraft pulp with xylanase 25 U/g of pulp with a retention time of 5 h at 10% consistency. The kappa number was reduced from 14.90 (kappa number of unbleached pulp) to 1.62. Li et al. reported that when wheat straw pulp was treated with the xylanase isolated from *Thermomyces lanuginosus *CBS 288.54, brightness was improved by 3.93% ISO [29]. Madlala et al. studied the effect of two xylanases (xylanase P and crude xylanase from *Thermomyces lanuginosus*) in the bleaching of kraft pulp and found that, xylanase P (at an enzyme dose 5 U/g pulp) increased the brightness of kraft pulp by 5.1% while xylanase from *Thermomyces lanuginosus* (at an enzyme dose 5 U/g pulp) increased 2.1% [8]. Kulkarni and Rao reported an increase in brightness of 2.5% and 21% reduction in kappa number by a xylanase (at an enzyme dose 10 U/g pulp) from *Bacillus *sp. NCIM 59 on unbleached bagasse pulp [2]. Angayarkanni et al. reported that xylanases from three fungi *Aspergillus indicus*, *Aspergillus flavus*, and *Aspergillus niveus* increased brightness of 42.0–45.0 ISO units from 19.83 ISO units and reduced kappa number of 5.0–6.8 from 18.60 of the kraft pulp [32].

To the best of our knowledge, this is the first paper on the thermo-alkali stable xylanase production from *Cellulosimicrobium* sp. MTCC 10645.

## Figures and Tables

**Table 1:** Effect of metal ions (1 mM, 5 mM, and 10 mM) on xylanase.

Metal ions	(Relative activity %)		
	1 mM	5 mM	10 mM
Ca^+2^	88.8	85.2	72.3
Hg^+2^	91.97	90.8	35.07
Fe^+2^	99.93	100.09	78.09
Cu^+2^	94.02	93.23	87.6
Mn^+2^	97.98	100	93.2
Na^+2^	112.8	109.8	99.8
Co^+2^	100.9	102.1	83.1
Pb^+2^	82.9	72.3	36.7
Zn^+2^	100.9	90.96	88.11
